# Effect of COVID-19 on Tuberculosis Notification, South Korea

**DOI:** 10.3201/eid2610.202782

**Published:** 2020-10

**Authors:** Nakwon Kwak, Seung-Sik Hwang, Jae-Joon Yim

**Affiliations:** Seoul National University College of Medicine, Seoul, South Korea (N. Kwak, J.-J. Yim);; Seoul National University Graduate School of Public Health, Seoul (S.-S. Hwang)

**Keywords:** coronavirus disease, 2019 novel coronavirus disease, COVID-19, SARS-CoV-2, severe acute respiratory syndrome coronavirus 2, respiratory diseases, zoonoses, viruses, South Korea, tuberculosis, bacteria, tuberculosis and other mycobacteria

## Abstract

After South Korea raised its infectious disease alert to the highest level in response to coronavirus disease emergence, tuberculosis notification during the first 18 weeks of 2020 decreased significantly from the same period for each year during 2015–2019. Adequate measures to diagnose, control, and prevent tuberculosis need to be maintained.

The first case of coronavirus disease (COVID-19) in South Korea was identified on January 20, 2020, and an outbreak from a church hastened widespread transmission throughout the country (*1*). On February 23, the government of South Korea raised the country’s infectious disease alert to the highest level and initiated vigorous infection control measures: establishing widespread diagnostic capacity, initiating local contact tracing, mandating physical distancing, and redesigning triage and treatment systems (*2*). While this alert level remains in effect, such measures could negatively affect other communicable diseases, such as tuberculosis (TB) (*3*). To investigate the effect of COVID-19 on TB diagnoses, we traced the number of notified TB cases in South Korea before and after the COVID-19 outbreak started and compared them with previous years, during which the burden of TB has been at an intermediate level.

We gathered the weekly number of newly notified TB cases for 2015–2020 from the Public Health Weekly Report released by the Korea Centers for Disease Control and Prevention. In South Korea, physicians and healthcare workers are required to report confirmed or clinically diagnosed TB to health authorities within 24 hours, irrespective of any previous history of TB treatment (*4*). The Public Health Weekly Report publishes the number of notified TB cases by province every week (*1*). In addition, the number of confirmed COVID-19 cases is posted daily on the Korea Centers for Disease Control and Prevention website (*1*).

We calculated the mean number of weekly TB notifications from the 1st through the 18th week of each year from 2015 through 2019. We also collected the weekly number of notified TB cases during the same period in 2020. We compared the number of cases before and after the highest alert level was declared (weeks 1–8 [before the COVID-19 outbreak began] and weeks 9–18 [after the COVID-19 outbreak began]). We estimated the change in the number of notified TB cases in 2020 after the COVID-19 outbreak started by comparing the latest numbers with those from previous years using a Bayesian structural time-series model (*5*). We used R statistical software version 4.0.2 (https://www.r-project.org) for all statistical analyses.

During 2015–2019, a mean number of 594 TB cases were notified weekly during weeks 1–8 and a mean number of 655 TB cases were notified weekly during weeks 9–18. In 2020, a mean of 498 TB cases were notified each week during weeks 1–8; the mean number of notifications during weeks 9–18 decreased to 390 cases/week. After COVID-19 began, TB notification decreased by 24% (121 cases/week; p<0.01 from the predicted number in 2020 based on a Bayesian structural time-series model) (Figure). In Daegu and Gyeongbuk Provinces, the epicenter of COVID-19 in South Korea, TB notification decreased by 23% (14 cases/week; p = 0.003). In other provinces, patterns were similar; TB notification decreased by 25% (112 cases/week; p = 0.001) after COVID-19 began (Table).

**Figure:**
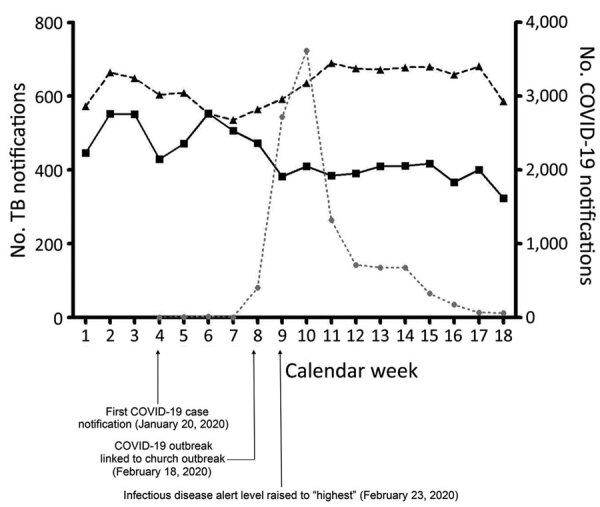
Mean weekly number of TB and COVID-19 case notifications in 2020 compared with the previous 5-year period, South Korea. Triangles indicate TB cases during 2015–2019; squares indicate TB cases during 2020; circles indicate COVID-19 cases during 2020. COVID-19, coronavirus disease; TB, tuberculosis.

**Table:** Weekly mean number of TB case notifications in 2020 compared with the previous 5-year period, South Korea

Location, calendar week†	Mean no. TB cases		Difference between actual and predicted cases after COVID-19 in 2020 (95% CI)
	2015–2019	2020	
All provinces			−121 (−165 to −86)
1–8	594	498	
9–18	655	390	
Daegu and Gyeongbuk Provinces			−14 (−24 to −3)
1–8	70	57	
9–18	77	46	
Other provinces			−112 (−153 to −79)
1–8	524	441	
9–18	576	344	

*COVID-19, coronavirus disease; TB, tuberculosis. †Weeks 1–8, before COVID-19 outbreak; weeks 9–18, after start of COVID-19 outbreak.

Our analysis demonstrated that the COVID-19 pandemic led to a decrease in TB notification in South Korea and that this reduction was not confined to the Daegu and Gyeongbuk Province areas. Although the number of TB cases in South Korea has decreased steadily since 2010 (*6*), the 24% decrease in TB notification after COVID-19 began is larger than that predicted by our time-series model.

The reduced number of TB notifications could reflect decreased transmission associated with physical distancing and the increased use of face masks. Recent analysis proposed that physical distancing could decrease transmission of TB by 10% in high TB burden countries (*7*). However, the 24% reduction in South Korea, which has an intermediate burden of TB, suggests the additional contribution of other factors. First, during the COVID-19 outbreak, interventions such as TB contact investigation and preventive therapy may have been deprioritized and delayed (*3*). Second, patients with newly developed respiratory symptoms could not visit chest clinics easily because those patients were redirected to COVID-19 screening clinics to prevent in-hospital transmission (*8*).

The negative effect of the COVID-19 outbreak on TB has not been confined to diagnosis. In South Korea, outpatient clinics and emergency departments have been temporarily closed after patients visiting the facility have been identified as having COVID-19 (*9*). Negative-pressure units also have been prioritized for COVID-19 patients (*2*). Overall healthcare use worsens during outbreaks of communicable diseases, as demonstrated by the 10%–23% decrease in emergency department visits, even for life-threatening conditions, after COVID-19 began, as reported in the United States (*10*).

In summary, we found that TB notifications decreased significantly with the surge of COVID-19 in South Korea. Adequate measures to diagnose, control, and prevent TB, a much older and more burdensome infectious killer than COVID-19, need to be maintained during this pandemic.
